# The forgotten patient: A psychological perspective on the implementation of bariatric surgery guidelines

**DOI:** 10.1002/osp4.670

**Published:** 2023-04-05

**Authors:** Lynne Johnston, Kacey Jackson, Charlotte Hilton, Yitka Graham

**Affiliations:** ^1^ Halley Johnston Associated Ltd Whitley Bay UK; ^2^ Golden Jubilee University National Hospital Scotland UK; ^3^ Helen McArdle Nursing and Care Research Institute Faculty of Health Sciences and Wellbeing University of Sunderland Sunderland UK; ^4^ South Tyneside and Sunderland NHS Foundation Trust Sunderland UK; ^5^ Lancaster University Lancaster UK; ^6^ Hilton Health Consultancy Derbyshire UK; ^7^ University of Florida Gainesville Florida USA; ^8^ University of Derby Derby UK; ^9^ Faculty of Psychology University of Anahuac Mexico Mexico City Mexico

**Keywords:** psychology, guidelines, patients, bariatric surgery

## Abstract

There is strong evidence demonstrating the impact of bariatric surgery on weight‐loss and comorbidity improvement. In the UK, there is specific guidance to facilitate the assessment of a person's suitability for bariatric surgery. This paper highlights the clinical reality of routinely implementing this guidance, supported by literature and the perspectives of practicing psychologists. The consequences of the implementation of clinical guidelines within the context of the typical biopsychosocial profile of those referred for bariatric surgery are discussed. The ramifications of a screening approach rather than a clinical formulation‐based approach to assessment, impact of a possible unconscious bias in commissioning and an overemphasis on a biomedical model approach to treatment are also presented. These contextual factors are argued to contribute to a population of “forgotten patients” that is, patients who have been assessed as not suitable for bariatric surgery, and thus “stuck” in their journey toward better health. For these individuals the only option left are energy balance only approaches, which are the very same approaches to weight‐loss and comorbidity improvement that have been attempted, often for many years. Not only have these approaches not resulted in weight‐loss and health improvement, they also fail to address the underlying psychological causes of obesity. Consequently, this lack of support means that patients continue to suffer from poor quality of life, with no clear pathway to improved health and wellbeing. This paper illuminates the clear gaps in weight management service provision, the implementation of guidelines in practice, and offers practical suggestions to reduce the unintended consequences of clinical guidelines for bariatric surgery.

## 1 INTRODUCTION

Bariatric surgery can lead to significant weight loss and improve obesity‐related comorbidities, such as Type 2 Diabetes.^1^ In the UK, bariatric surgery is recommended by the National Institute for Health and Care Excellence (NICE)^2^ as a treatment option for patients with a BMI of 40 or above, or 35 and above with obesity‐related comorbidities (e.g., Type 2 Diabetes), and as a first line treatment for those with a BMI above 50, and those with uncontrolled diabetes.^2^ Prior to being considered for bariatric surgery, people living with obesity are required to have attempted non‐surgical weight‐loss interventions, such as dietary support; physical activity; and use of medication (e.g., Orlistat®).

Patients seeking bariatric surgery should receive “intensive management” in a Tier 3 service (weight management program that supports adults with severe and complex obesity to lose weight through a range of interventions such as psychological approaches and dietary changes)^2^ and commit to the requirement of long‐term follow‐up. However, the operational definition of what “intensive management” comprises is lacking. Owing to acknowledged variation in UK commissioning for weight management services, there are disparities in terms of interpretation as to “intense management” and what this entails, meaning many people living with obesity in Tier 3 services may not receive an equitable level of care and support.

UK guidelines recommend that each Tier 3 Weight Management Service should include a minimum of a Clinical Psychologist, Dietitian, Nurse, Pharmacist, Physician, and Surgeon.^3^ The guidelines stipulate that bariatric surgery should *only* take place if a multi‐disciplinary team (MDT) can provide psychological support before and after surgery. Crucially, this should include a comprehensive pre‐operative psychological assessment to highlight psychological or clinical factors that may negatively impact the patient.^3^, ^4^

Living with a bariatric‐surgically altered body requires life‐long lifestyle changes (e.g., eating, alcohol, activity habits). Clinical management of long‐term multifactorial behavioral changes^5^ can be challenging.^6^ Evidence suggests that follow‐up support from clinical psychology is often crucial to post‐surgical health behavior change.^7^ Immediately following bariatric surgery, weight loss is rapid,^8^ particularly in the first 2 years, slowing down, often plateauing, and weight gain often occurs at this time.^9^, ^10^, ^11^ Post‐bariatric surgery, poor psychological outcomes can include an increased risk of suicide, self‐harm, instability in mental health, and addiction transfer.^9^, ^10^, ^11^

Currently, guidelines available for clinicians working within Bariatric Psychology include NICE Obesity Management guidelines,^2^ British Obesity and Metabolic Surgery Society (BOMSS) Tier 3 Commissioning guidelines,^3^ and BOMSS‐endorsed psychology guidelines.^12^ However, it is proposed that the practical reality of implementing these guidelines has not been previously published, resulting in missed opportunities to share learning from clinical practice. With the increasing number of people living with obesity being referred for surgery,^13^ practice‐based evidence to inform service development and optimal patient care is helpful to inform future commissioning decisions for Tier 3 Weight Management Services.

The aim of this review is to offer a pragmatic discussion of the implementation of the current bariatric guidance in routine clinical psychology practice. The service context is situated within a Tier 3 Service in the North‐East of England, where assessment of over 1000 patients referred for bariatric surgery underpins the evidence posited in this paper.

### 1.1 The relationship between living with obesity and mental health difficulties

Evidence shows a consistent biopsychosocial relationship between people living with obesity and mental health difficulties.^3^, ^14^, ^15^, ^16^ People who seek bariatric surgery can often present with complex psychopathology (e.g., depression, eating disorders, substance misuse, & poor life quality).^17^, ^18^, ^19^ Correlational studies fail to conceptualize the complexity due to a focus on a limited number of demographic variables and presenting “symptoms” investigated in relative isolation.^20^

Existing studies show a lack of attention to patients who could potentially benefit from bariatric surgery but whom have been deemed ineligible. There is emergent literature regarding the socioeconomic characteristics of patients who do proceed to surgery, but no research from the UK regarding those who do not progress.^21^, ^22^, ^23^ Bariatric patients with a BMI above 50 can present, at initial referral, with various co‐existing difficulties (e.g., poor physical & mental health, eating disorders, and substance abuse) which can further maintain ongoing presenting problems.^24^, ^25^ Compared with vast evidence of the etiology and treatment of obesity, there is a paucity of studies about how the implementation of treatment guidance operates within routine psychology practice and service delivery. This presents the challenge of the over emphasis of evidence‐based practice at the expense of practice‐based evidence.

### 1.2 The emphasis on a medical model approach to psychological difficulties encourages a “one size fits all” approach to interventions

Commonly, there is a focus on a biomedical model of care in services working with people living with obesity. By contrast, a biopsychosocial model encompasses a wider perspective to include psychological and social factors influencing health and illness. A binary focus on biological and physical aspects alone does not help to fully account or explain for outcomes following bariatric surgery.^26^ In primary and acute health settings there is commonly a focus on *symptoms* rather than *causal* factors. Thus, when patients living with obesity are referred to an acute service for bariatric surgery, the underlying causal factors are rarely highlighted, screened for, or addressed. Patients with a long established and untreated trauma, loss, or attachment difficulty may present with an increased number of medical and psychological comorbidities.^27^ If these conditions remain untreated this can impact negatively on post‐surgical outcomes and mental health stability, meaning an emphasis placed on a biomedical “*one size fits all*” approach is not appropriate for a cohort of patients with known complex psychopathology.

There is a common misconception that the bariatric surgery itself will “treat” the psychological presenting “symptoms” (e.g., depression), which is a potentially damaging perspective which fails to acknowledge or understand the complexity of the causal and maintaining psychological mechanisms which underpin complex psychological difficulties. In clinical psychology, an “iceberg” analogy is often used to conceptualize the limitations of a symptom‐focused biomedical approach to assessment and treatment planning. The tip of the iceberg is the part that is visible (analogous to the presenting symptoms), with the causal factors which often remain invisible (underwater). This represents the predisposing, precipitating, and co‐maintaining factors of the visible symptoms. The co‐maintaining relationships between living with obesity and living with mental health difficulties are often complex and multifactorial, justifying why a biopsychosocial formulation‐based approach offers significant clinical utility.^28^ The greater the level of complexity in a client's presentation, the more limited a *one size fits all* approach is to assessment, formulation, and treatment planning approaches within healthcare.^29^

### 1.3 What are the increased risks for those who have had bariatric surgery and why?

A significant proportion of the adults living with obesity who attend Specialist Weight Management Services (Tier 3) in the UK have experienced childhood adversity (e.g., abuse, familial mental illness, trauma, & family conflict),^30^, ^31^, ^32^, ^33^ including childhood obesity, with girls being more sensitive to obesity‐related effects.^34^ The exact reasons for this are unclear, but this may be due to the disproportionate level of physical and sexual violence perpetuated toward women in both childhood and adulthood compared to men. For example, approximately 736 million women globally (almost one in three) have been subjected to violence at least once in their life.^35^ Further, childhood sexual abuse is cited as having the greatest impact on living with childhood obesity in comparison to other Adverse Childhood Experiences (ACEs), with multiple ACEs predicting increased risk.^34^ Patients with unresolved trauma are more likely to present after bariatric surgery with difficulties, for example, increased alcohol use; unhealthy eating behaviors; poor weight‐loss; and increased suicide risk.^36^, ^37^, ^38^, ^39^ The use of food as an emotional regulatory strategy, and as a method of self‐soothing has been postulated as an explanatory mechanism.^32^

Although there are improvements in cognitive function post ‐bariatric surgery for those with pre‐surgical severe and complex obesity, there is a correlation between elevated levels of disinhibition and lower levels of restraint with poor compliance with post‐surgical dietary recommendations and suboptimal weight loss 2 years after surgical intervention.^40^

The use of alcohol or substances as a maladaptive coping strategy pre‐ and post‐surgery can result in poorer weight loss outcomes post‐surgery, and increased risk to patient's physical and mental health.^41^, ^42^ Studies confirm that those with a higher prevalence of post‐operative alcohol and substance use also consumed excessive levels of alcohol or substances prior to bariatric surgery.^41^, ^42^ The use of alcohol or substances as an emotional regulatory strategy during times of difficulties has been shown to be predictive of post‐surgical alcohol use.^41^, ^42^, ^43^ Patients who have had Roux‐n‐Y gastric bypass surgery have been shown to have increased rates of alcohol disorders and substance abuse 7 years after surgery.^44^ It is imperative to ensure that patients have adaptive and robust alternative coping strategies in place for distress tolerance as part of their preparation for bariatric surgery.

Following bariatric surgery, patients can experience an improvement in their mental health and psychosocial functioning, although this is not universal. Some patients experience different psychosocial concerns, such as maladaptive eating behaviors; body image concerns (e.g., excess skin); substance abuse; suicidal thoughts; self‐harm; transfer of addiction; lack of social support; and completed suicide.^45^ Insight into the importance of psychological contraindications prior to bariatric surgery has been provided by investigating post‐operative psychosocial concerns and self‐injury.^45^ Pre‐operative suicidal ideation and self‐harm behavior is the most significant predictor for post‐surgical self‐harm and completed suicide.^46^ Self‐harm within the 2 years prior to bariatric surgery is a significant predictor for self‐harm in the 2 years post‐surgery.^47^ Patients who experience impairments or worsening of their quality‐of‐life post‐surgery often experience feelings of disappointment and failure in relation to their expectations.^48^ A systematic review of 14 studies by Gill et al. found there were significant reductions in depressive symptoms in 13 studies,^49^ but the timeframe of the studies was limited to 2–3 years, which is often referred to as the “honeymoon” period after bariatric surgery. There are a lack of studies examining mental wellbeing in the longer‐term after bariatric surgery, which are needed to build a comprehensive understanding of patients outside this initial timeframe. Disordered eating and distorted body image have been shown to be associated with an increased suicide risk in those patients who develop (or re‐develop) loss of control over their eating behaviors following bariatric surgery (including subjective binge eating & self‐induced vomiting).^48^ Substance or alcohol abuse after surgery have also been associated with increased risk of self‐harm and suicidality after bariatric surgery.^50^ Weight re‐gain, metabolic changes, recurrence of comorbid diseases, and unrealistic expectations have all been associated with suicidality and self‐harm behaviors post‐surgery.^51^, ^52^ The importance of pre‐surgical psychological assessment and evidence‐based interventions needs to be clearly articulated and embedded into policy and practice within bariatric surgery.

The psychological consequences of bariatric surgery can be significant, reinforcing the importance of pre‐surgical psychological assessments and the critical role of the clinical psychologist in pre‐, peri‐, and post‐operative care of the bariatric patients. Given the current variability in psychological provision across the UK, and the increasing demand for bariatric surgery provision, significant investment in psychological provision is urgently required.

## 2 THE CLINICAL REALITY OF THE IMPLEMENTATION OF BARIATRIC GUIDELINES

### 2.1 What are the BOMSS Tier 3 Commissioning guidelines based on and what is the clinical reality of their implementation?

In the UK, the British Obesity and Metabolic Surgery Society (BOMSS) Tier 3 Commissioning guidelines^3^ state that part of the clinician's role should be to appropriately facilitate bariatric surgery for patients who fulfill the BMI thresholds stated within the NICE guidelines.^2^ The justification for a psychological assessment for bariatric surgery is to identify patients that have unrealistic expectations regarding bariatric surgery. This may also include the impact surgery will have upon their life and weight loss; psychological contraindications for bariatric surgery; and those who require additional support from psychology prior to the surgery. The assessment should also highlight any stressors which may have a negative impact upon patients' engagement with the post‐operative requirements of self care; previous stressors which are associated with the development of living with obesity, weight regain following weight loss and patients requiring psychological support in the longer term.^3^

The BOMSS guidelines utilize Steven et al.’s^53^ traffic‐light analogy in outlining those patients who may be unsuitable for bariatric surgery (*red*), those who require additional support (*amber*), and those who are potentially suitable (*green*). Unsuitable patients may include those with unstable psychosis; active substance misuse/alcohol dependence; severe learning disability; dementia; severe personality disorder; and current non‐adherence to treatment. Those who are classified as “*amber*” require extra support before surgery. Steven et al.^53^ cites examples to include those with untreated or unstable mental health presentations; active alcohol or substance misuse; a history of or an active eating disorder *without psychological treatment*; self‐harm or suicidality in the past 12 months; or recent significant life events. “*Green*” patients are deemed suitable for surgery and are required to demonstrate appropriate motivation and expectations; good understanding of the procedure and outcome; good understanding of the impact of diet; a regular balanced diet; and proven adherence to treatment.

Although the traffic light system can be a useful guide, the reality is that often, clinical decision‐making within an MDT can be more complex. The quality of the decision‐making is only as good as the “data” upon which the data is based, and this is complicated by self‐reported data by the patient. In our clinical experience, patients can feel that bariatric surgery is their last chance to achieve sustained weight loss, so it is not uncommon for information to be withheld or minimized by patients in order to enhance the likelihood of a progression to surgery. This may be exacerbated within Services where the clinical psychologist is placed into the position of the “gatekeeper” to surgery. From a surgical risk assessment and safety point of view, an MDT requires accurate, up‐to‐date, and factually correct information regarding a patient physical health status. The risks of operating on a patient without this information are significant, yet, psychological screening informed largely by self‐reported information remains a widely accepted practice. The importance of obtaining accurate and up to date information regarding a bariatric patient's psychological health status has historically not been fully acknowledged and prioritized.^12^ By providing an in‐depth psychological assessment based on accurate reports of a patient's mental health background, including reports from their GP and all mental health professional/services, offers the potential for a more comprehensive assessment to be conducted. This is not routine clinical practice within bariatric psychology in the UK; consequently, a significant risk is that some patients may proceed to surgery when they are psychologically unsuitable thereby increasing the risk of post‐surgical complications.

In our clinical experience, it is often this group of patients who can subsequently present with post‐surgical “medically unexplained” symptoms which may lead to multiple failed medical tests and interventions. It is imperative to recognize that in cases where something is “medically unexplained” there may well be a underlying psychological explanation,^54^ whereby unresolved psychological distress may manifest as physical symptoms (e.g. “unexplained abdominal pain” is relatively common in post‐bariatric surgery patients).^55^ Often a standardized approach to screening measures and manualized treatment protocols do not allow the level of individualization needed to work with complexity. Alternatively, the use of a collaborative biopsychosocial formulation‐based approach does allow a greater detail of information to be utilized and this is more consistent with a patient‐centered approach to a clinical psychology‐based intervention.

The BOMSS guidelines used previous literature to identify psychological contraindications, for example, approximately 15% of bariatric patients were declined surgery with the most common rationale being significant psychopathology (e.g., psychosis, bipolar disorder); untreated depression; and a lack of understanding about post‐operative requirements and the risks associated with bariatric surgery.^56^ The most common reasons for surgery denial were high levels of psychological distress, binge eating, and a history of alcohol misuse, and in most cases, the psychological stress pre‐dated the development of obesity.^56^

Context‐specific eating disorders, including Binge Eating Disorder (BED) and Night Eating Syndrome (NES) are more common in a bariatric surgery seeking population,^57^ e.g., the prevalence of disclosed and diagnosed BED in the general population is approximately 2.6% and 21.3% in the bariatric surgery‐seeking population.^58^, ^59^ This confirms the clinical reality of a known over‐representation of patients presenting to a bariatric pathway who meet the Diagnostic and Statistical Manual of Mental Disorders Fifth Edition (DSM‐5) criteria for BED.^60^ In reality, the “known” percentage is likely to be a serious underestimation of the true extent of the presenting clinical picture, which may be due to the highly secretive nature of eating disorders, and the shame and internal stigma associated with eating disorders in those living with obesity. This underestimation may be exacerbated by poor quality screening and assessment for obesity‐related eating disorders within primary care (e.g., BED, NES)^59^, ^61^, ^62^, ^63^ and within specialist weight management services at both Tier 2 and Tier 3 in the UK.^64^, ^65^, ^66^

The NICE guidelines for eating disorders^67^ state that eating disorders should be treated *before* addressing weight management concerns. However, the NICE Obesity Management guidelines suggest bariatric surgery is a first line treatment for those with a BMI of 50 or above.^2^ Given that obesity‐related eating disorders are not routinely screened for within all Tier 2 and Tier 3 Services (or voluntarily disclosed by patients) it is unsurprising that so many services prematurely focus on obesity treatment at the expense of the eating disorder. As an untreated eating disorder is a likely contraindication for bariatric surgery,^3^, ^12^ this generates the dilemma for patients regarding whether to make a full disclosure of underlying causal factors or not. If a known eating disorder is reported in those living with obesity, the next dilemma is which service is specifically commissioned to work with these patients and what the specific treatment recommendations are?

Bariatric psychology provision is affected by current commissioning practices in several ways. For example, at the time of writing there are no commissioned specialist psychological services in the North‐East of England for the treatment of patients presenting with an eating disorder and living with obesity, meaning there is a focus on specialist eating disorder services is for patients who present with anorexia and complex bulimia. However, eating disorders associated with higher BMI's are more prevalent^68^, ^69^, ^70^ with one study showing that AN accounted for 8% of eating disorder cases, whereas BED accounted for 22%.^71^ There are also lengthy delays in treatment for patients presenting with Bulimia Nervosa (BN) and BED in comparison to AN with noted low treatment rates from a mental health specialist for people with BN (25%) or BED (21.9%), despite high rates of General Practice attendance within the previous 3 months (72.2% & 90.6%).^72^

In our clinical experience, patients living with obesity who also meet DSM‐5 criteria for BED are usually referred via Primary Care, for support with weight loss rather than for the treatment of their disordered eating. This premature focus on weight loss is inconsistent with NICE (2017) eating disorder guidelines and may exacerbate the underlying eating disorder etiology by reinforcing a focus on “dieting” and an over‐evaluation of weight and shape. This is the rationale for evidence‐based treatments for eating disorders (e.g., CBT‐e) recommending a focus on treating the eating disorder first *before* focusing on weight‐related treatments.^2^, ^67^, ^73^ The reasons for this apparent discrepancy in commissioning of services remain unclear. One theory may relate to mortality data and the listing of AN as a contributory causal factor on a death certificate. In our clinical experience, those patients who die within the context of an untreated eating disorder and who are living with obesity typically have the primary cause of death (e.g., cardiac arrest) listed with obesity as a “secondary” causal factor.

One theoretical focus within a bariatric population suggests that excess eating is a habitual coping mechanism for emotional distress, with an increased prevalence in those less able to tolerate distress.^36^ Whilst binge eating behaviors may result in a temporary reduction in low mood or distress, the subsequent feelings of guilt, shame, and/or self‐disgust can reinforce further binge eating behaviors. If these habitual maladaptive coping responses are not addressed prior to bariatric surgery, then there may be an increased risk of post‐surgical complications. For example, the patient may replace food with an alternative (maladaptive) behavioral response in an attempt to cope with their low distress tolerance (e.g., loss, attachment issues). These often remain as an unresolved causal mechanism for the resultant maladaptive behavioral response. A related consideration is that people diagnosed with an Borderline Personality Disorder (BPD) are over‐represented in a bariatric population.^74^, ^75^, ^76^ Chronic feelings of emptiness, strong emotional responses and impulsivity are all associated with a BPD diagnosis with binge eating specifically listed as one example of a behavior characterizing impulsivity in the list of DSM‐5 diagnostic criteria.^60^ Therefore, it is conceptually unsurprising that maladaptive emotional regulation strategies are significant mediators of the relationship between BPD and dysfunctional behaviors, one of which is binge eating.^77^, ^78^ Based on our clinical experience and DSM‐5 criterions for both BPD and BED, there is clear overlap between the two presentations (i.e., chronic feelings of emptiness, strong emotional response, impulsivity). Clinically, patients in a bariatric pathway may often present with BPD and BED behaviors but with no formal diagnosis of either per se. Many of these patients have had little prior contact and involvement with mental health services but they can often have a long history of “weight cycling” and longstanding maladaptive coping behaviors in response to low distress tolerance (e.g., via eating, alcohol, or recreational drug use behaviors).

Existing guidelines are open to interpretation and may risk placing the clinical psychologist in a “gatekeeping” role. This can be detrimental as it may encourage patients to minimize their difficulties to favorably navigate the assessment stages in order to “qualify” for surgery rather than working with the psychologist to address the underlying issues that have caused and maintained their weight management difficulties in the first place. This is exacerbated in service structures that include an assessment‐only role for bariatric psychology, further adding confusion of who is commissioned to work with patients living with obesity and presenting with a context specific eating‐disorder (e.g., BED/NES).

There is an ongoing debate as to how psychological risks associated with bariatric surgery should be balanced with the physical and psychological health risks associated with not proceeding with surgery and who should be responsible for this decision‐making process.^79^ The clinical reality is that some patients may be excluded from surgery due to untreated contraindications; often there are no specific services commissioned to work with these patients, so these conditions remain untreated, with continued negative impact on quality of life and potentially increased risk of harm.

### 2.2 What are the BOMSS‐endorsed psychology guidelines and what are the implications of implementing these guidelines?

In 2019, Ogden et al. published the BOMSS‐endorsed psychology guidelines for bariatric surgery.^12^ These guidelines suggest a three‐step service model for psychological support before and after bariatric surgery. The authors argue that the first step should be available to all patients within a bariatric pathway and include online information. The second step is a referral into a group‐based workshop provided by “up‐skilled” allied health professionals. However, no details are provided regarding the minimum standards, training and supervision requirements and arrangements for the “up‐skilled” allied health professionals as this is beyond the scope of the guidelines. The implicit recommendation is that interventions are conducted under the care and coordination of an experienced clinical psychologist. The third step involves a referral to a clinical psychologist (if there is no resolution after Step two, or if the significance of the issues requires further assessment). The step three assessment is undertaken by a suitably qualified clinical psychologist who then has the choice of either referring the patient on to another external service (it is unclear which/why) or a 1:1 treatment intervention with the clinical psychologist working within the bariatric surgery team.

Whilst the BOMSS psychology guidelines provide a useful starting point, several challenges arise in the interpretation and implementation of these guidelines in clinical practice. For example, it is unclear who undertakes the initial “psychological triage screening” and what specifically the screening should include and why (i.e., does this cover all potential identified issues from in the Ogden et al. guidelines?). Clinical experience would suggest that this work could be undertaken by a psychologist (e.g., assistant psychologist) trained and supervised by an experienced bariatric clinical psychologist but with the current variance in practice and workforce shortage it is unknown what happens in practice, why and with what impact on patient care.

There have been significant cuts to the funding of clinical psychology training places in the UK since 2004 which has impacted on provision of psychological services.^80^ Failure to address the shortage of UK clinical psychologists in bariatric surgery is likely to impact on the quality and availability of the pre‐ and post‐operative bariatric surgical care provided. Specifically, the risk is that more patients will experience significant difficulties post‐surgery if they have proceeded to surgery without adequate psychological preparation and support before surgery. There may be a risk of more patients (who could potentially benefit from surgery) being excluded from surgery due to untreated risk (e.g., eating disorders) in line with the correct implementation of current guidelines. Our psychological service provision has shown, these issues need to be address *within* a bariatric psychology service to optimize continuity of care.

It is imperative to have a clear understanding of the relationship between living with obesity and living with mental health difficulties to fully utilize and understand the role of clinical psychology in the pre‐, peri‐, and post‐operative care of those who seek bariatric surgery. A screening process primarily focuses upon issues at a *symptom* level only and, a patient's presenting symptoms will likely not capture the underlying causal and co‐maintaining factors (i.e., trauma, loss, attachment) which may continue to impact upon the patient in a detrimental way. A further question remains as to *when* the screening should take place, for example, should it be part of the initial assessment, or should it only be conducted as part of a specific referral process to clinical psychology? This adds to the debate about whether a structured referral form should be implemented prior to the patient entering the overall bariatric pathway to screen for any psychological contraindications and who is clinically responsible for managing this process.

It is unclear who monitors the uptake and implementation/progress of Step 1, and how this is specifically assessed before a referral is made to Step 2. The group‐based workshops also lack specificity. For example, it is unclear whether these group sessions are designed as therapeutic or psycho‐educational groups and what specifically these groups would be aiming to target and why (e.g., binge eating; night eating; distress tolerance; self‐worth; behavioral avoidance; behavioral regulation; etc). Given the complexity of this patient group and the nature of the material delivered, we would argue that the groups should be designed and co‐delivered by a suitably qualified and experienced clinical psychologist. It is questionable whether an “up‐skilled allied health professional” is best placed to deliver these groups alone as they are unlikely to be fully equipped to identify, assess, and formulate psychological risk‐related issues and underlying causal factors (e.g., trauma) which require a Step 3 intervention. The BOMSS guidelines may be interpreted as stating that patients could have no direct contact with a qualified and experienced Clinical Psychologist until Step 3. Often the perceived benefit of a stepped‐care approach to services is cost savings; yet a key consequence is the significant risk of a premature focus on symptoms at the expense of a more complete formulation of causal and maintaining factors.

Our psychological service provision has shown the majority of people referred to a bariatric pathway can be classified according to the Steven's model^53^ as *amber* or *red*, both of which would require a direct referral to a Clinical Psychologist at a Step 3 level.^81^ Therefore, it is unclear in these guidelines how the stepped care model would maximize the use of this scarce resource in the current climate. A premature focus on symptoms alone increases the risk of too many patients going through Step 1 and Step 2 first, which may be an inefficient use of time and funding when the complex causal factors could have been identified at an earlier stage within psychology. It would be useful for the model to have a direct pathway from the “identification of issue” directly to Step 3 with additional recommendations of how to measure progress and who should be undertaking assessment and referral processes.

There are two potential outcomes from an assessment by a Clinical Psychologist according to BOMSS guidelines, either a 1:1 intervention with a Clinical Psychologist located within the bariatric service or a referral to an external service as appropriate. In practice, the referral to an external service is both limited and fragmented because generally, the only option is an onward referral to a generic primary care mental health service [e.g., Improving Access to Psychological Therapies (IAPT) Services]. In our clinical experience, IAPT services are *not* commissioned to work with the level of complexity seen within bariatric services and they may be only commissioned to offer up to 12 sessions per treatment episode. This reiterates an important commissioning question regarding where the work needs to be conducted for those patients who are assessed as unsuitable for surgery due to untreated contraindicators.

It is acknowledged that the BOMSS‐endorsed psychology guidelines are designed with flexibility in mind given the variance in practice across the UK, yet the potential unintended consequences of this are that they may invite further variance and interpretation in practice which may impact on patient care. A result of this variance in practice is a lack of parity in the provision of Tier 3 and Tier 4 Specialist Weight Management/Bariatric Psychology Services across the UK. The BOMSS‐endorsed psychology guidelines clearly state that all Tier 4 patients who are referred to a bariatric pathway for consideration for revision surgery should be re‐assessed by a Clinical Psychologist.^12^, ^82^ The responsibility for managing this process is not specified as a result of discrepancies in clinical practice. The lack of funding provision for people living with obesity *and* an eating disorder exacerbates the difficulties for the patients contraindicated for bariatric surgery and often these patient's treatment needs remain unaddressed because their needs fall between gaps in commissioned service provision.

It is vital that psychological guidance is clear in order to be implemented correctly to meet the complexity the patients within weight management services present with. Patients within Tier 3 Weight Management services often present with a complex mix of *symptoms*, which can be conceptualized using a collaborative, formulation‐based approach.^83^ This approach can help patients to understand the connections between the biological, social, and psychological (e.g., trauma, complex bereavement, attachment difficulties) *causal* factors.^83^ Clinical Psychologists are specifically trained in case formulation as a productive way of engaging and working with clients^83^, ^84^, ^85^; this may be one of the reasons why the current guidelines emphasize the need for representation from a clinical psychologist with expertise in bariatric/specialist weight management services.^2^, ^3^, ^12^, ^67^, ^81^, ^82^

## 3 INTERNATIONAL GUIDELINES

This review focuses on implementation of BOMSS guidelines and experiences of clinical psychologist working in bariatric surgical services in the UK. It is important to be aware of other international guidelines pertaining to the psychological care of bariatric surgical patients. The updated IFSO/ASBMS guidelines discuss the higher rates of suboptimal psychopathology in bariatric surgical candidates and to assess candidates' suitability to cope with postsurgical adaptations including lifestyle, body image and surgery itself, and to identify external stressors such as food insecurity and housing. The AACE/ACE/TOS/ASMBS/OMA/ASA guidelines recommend comprehensive and formal psychological assessment prior to surgery, undertaken by a qualified behavioral health professional with specialist knowledge of obesity, eating disorders and an understanding of bariatric surgery and its impact on patients,^86^ but do not mention a stepped approach as recommended in the BOMSS guidelines.^12^ Attention to assessment of environmental, familial, and behavioral factors and risk for suicide should be required for all patients prior to surgery, and any patient with a diagnosed or suspected psychiatric illness, or substance abuse or dependence should have a formal mental health evaluation as part of presurgical assessment. Similar, but more strict than advice in BOMSS guidelines,^12^ are that RYGB, SG and high‐risk groups should eliminate alcohol consumption due to impaired alcohol metabolism and risk of alcohol‐use disorder postoperatively.^86^ All three guidelines state that patients should be assessed for their ability to incorporate behavioral and nutritional changes and requirements before and after any bariatric procedure.^12^

## 4 KEY CONSIDERATIONS AND CONSEQUENCES OF THE IMPLEMENTATION OF CLINICAL GUIDELINES: IMPLICATIONS FOR PRACTICE, RESEARCH, AND POLICY

A significant number of patients referred to bariatric services do not proceed to surgery.^12^, ^81^ A proportion are deemed to be contraindicated on psychological grounds.^56^ However, there is a dearth of published information to describe who these patients are and why, specifically they have been identified as contraindicated for bariatric surgery.

### 4.1 The “forgotten patient”: Implications and recommendations

Given the lack of published bariatric literature in this area, and the lack of access to alternative treatment provision, these people are reflective of the “forgotten patient” within the weight management care pathway. The lack of available literature may be owing to data not being routinely collected in practice about those who do *not* proceed to surgery. The existing literature focuses on those who do proceed to have surgery, and those who return to the bariatric pathway post‐surgery presenting with complications and/or “medically unexplained symptoms.”^29^ Highlighting the specific treatment needs of those who are currently excluded from surgery on psychological grounds may help to inform commissioning decisions by highlighting gaps in service provision. Currently most bariatric funding is allocated to the patients surgical and medical costs. However, when we consider the critical role of psychology in assessment, formulation, and treatment that we have presented here, it is clear that insufficient funding allowance is provided for the psychological aspects of bariatric surgery. Furthermore, it is reasonable to suggest that there may be evidence of unconscious bias in the commissioning of weight‐related services with an over‐emphasis on commissioning of services for patients diagnosed with disordered eating resulting in low weight (i.e., anorexia nervosa) rather than for those who are living with obesity.

Patients who are assessed as contraindicated may well be suitable for bariatric surgery in the future if their treatment needs are addressed. However, as outlined, many of these individuals do not receive timely and effective treatment due to lack of access and/or formal pathways to suitable treatment provision. This is consistent with our clinical experience where we know patients often present as complex, marginalized, stigmatized, and with a long history of failed provision. The patients are often deemed unsuitable for Primary Care Mental Health Services, and Regional Eating Disorder Services, and because they are not viewed as presenting with an urgent risk or in need of a coordinated care‐plan they tend to be deemed unsuitable for Secondary Care Mental Health Services. These patients can find themselves being referred between mental health and bariatric services, and they may be repeatedly declined bariatric surgery from different bariatric units due to untreated psychological risk factors. Yet at the same time, they are unable to access evidence‐based treatment in line with clinical treatment guidelines.^2^, ^3^, ^12^, ^67^

## 5 CONCLUSION

In our clinical experience, there are clear gaps within bariatric psychology service provision. There is a need for the development of clearer clinical guidelines for psychological assessment and treatment within bariatric services. Further, there should be continued investigation into the lack of available interventions for patients presenting to a bariatric pathway with complex mental health difficulties. UK‐based research studies are needed within bariatric psychology to assess and evaluate the specific treatment needs of patients who have been deemed unsuitable for surgery on psychological grounds. Such studies would contribute to informing the process of responding ‐ both clinically and financially, to the urgent need to better support these forgotten patients.

## AUTHOR CONTRIBUTIONS

Kacey Jackson scoped the review under direct clinical guidance and supervision of Lynne Johnston. Lynne Johnston, Kacey Jackson, Charlotte Hilton and Yitka Graham contributed to writing up the manuscript.

## CONFLICT OF INTEREST STATEMENT

The authors have no conflict of interest to declare.
